# Quinine Hypodermatically for Malaria

**Published:** 1893-08

**Authors:** 


QUININE HYPODERMATICALLY FOR MALARIA.—The hypodermatic use of quinine and urea for malaria is in a fair way of being revived. Cohen (*Polyclinic*, p. 66) advocates the bimuriate of quinine and urea, a plan that was strongly endorsed in 1887 by Dr. R. P. Talley, of Texas. Ten or fifteen grains of the salt are dissolved in twenty to thirty minims of boiled or sterilized water and injected deeply into the subcutaneous tissues. Three injections are made during the first seven days of treatment and two injections the second, after which arsenic in some suitable form is administered for two or three weeks. Dr. Cohen recommends that the syringe be thoroughly emptied before it is withdrawn, and the subsequent painting of the puncture with tincture of iodine.

The method is similar to that practiced by Dr. George Dock, while located at Galveston, although Dock employed the ordinary sulphate with sufficient acid to produce a solution with water; he was particular not to inject more than from six to eight grains at one time, but several punctures could be made, thus enabling the medicine to be administered in sufficient quantity, and as a rule but one or two injections were required. In a large number of cases operated upon, Dr. Dock made a preliminary examination of the blood and demonstrated the presence of the *plasmodium malariæ*.

